# Modulation of Cellular NAD^+^ Attenuates Cancer-Associated Hypercoagulability and Thrombosis via the Inhibition of Tissue Factor and Formation of Neutrophil Extracellular Traps

**DOI:** 10.3390/ijms222112085

**Published:** 2021-11-08

**Authors:** Wa Cao, Meng-Yu Zhu, Seung-Hoon Lee, Su-Bin Lee, Hyung-Jin Kim, Byung-Ouk Park, Cheol-Hwan Yoon, Dipendra Khadka, Gi-Su Oh, Hyeok Shim, Tae-Hwan Kwak, Hong-Seob So

**Affiliations:** 1 Center for Metabolic Function Regulation and Department of Microbiology, School of Medicine, Wonkwang University, Iksan 54538, Jeonbuk, Korea; caowa1990@163.com (W.C.); zhumengyu6@gmail.com (M.-Y.Z.); liveleee@gmail.com (S.-H.L.); lmj1094@hanmail.net (S.-B.L.); 2 NADIANBIO Ltd., R201-1, Business Incubation Center, 460 Iksan-daero, Iksan 54538, Jeonbuk, Korea; enz94@nadianbio.com (H.-J.K.); bopark@nadianbio.com (B.-O.P.); yooncheolhwan@nadianbio.com (C.-H.Y.); dpen.khadka15@gmail.com (D.K.); echemogs@gmail.com (G.-S.O.); bioalpha140@nadianbio.com (T.-H.K.); 3 Internal Medicine, School of Medicine, Wonkwang University, Iksan 54538, Jeonbuk, Korea; mdshim13@naver.com

**Keywords:** cancer-associated thrombosis, NAD^+^, dunnione, tissue factor, NETs

## Abstract

Cancer-associated thrombosis is the second-leading cause of mortality in patients with cancer and presents a poor prognosis, with a lack of effective treatment strategies. NAD(P)H quinone oxidoreductase 1 (NQO1) increases the cellular nicotinamide adenine dinucleotide (NAD^+^) levels by accelerating the oxidation of NADH to NAD^+^, thus playing important roles in cellular homeostasis, energy metabolism, and inflammatory responses. Using a murine orthotopic 4T1 breast cancer model, in which multiple thrombi are generated in the lungs at the late stage of cancer development, we investigated the effects of regulating the cellular NAD^+^ levels on cancer-associated thrombosis. In this study, we show that dunnione (a strong substrate of NQO1) attenuates the prothrombotic state and lung thrombosis in tumor-bearing mice by inhibiting the expression of tissue factor and formation of neutrophil extracellular traps (NETs). Dunnione increases the cellular NAD^+^ levels in lung tissues of tumor-bearing mice to restore the declining sirtuin 1 (SIRT1) activity, thus deacetylating nuclear factor-kappa B (NF-κB) and preventing the overexpression of tissue factor in bronchial epithelial and vascular endothelial cells. In addition, we demonstrated that dunnione abolishes the ability of neutrophils to generate NETs by suppressing histone acetylation and NADPH oxidase (NOX) activity. Overall, our results reveal that the regulation of cellular NAD^+^ levels by pharmacological agents may inhibit pulmonary embolism in tumor-bearing mice, which may potentially be used as a viable therapeutic approach for the treatment of cancer-associated thrombosis.

## 1. Introduction

Cancer is commonly associated with hypercoagulability and a high risk of thrombosis, which make it the second leading cause of mortality in patients with cancer [1]. Anti-cancer treatments, such as surgery, chemotherapy, angiogenesis inhibitors, and central venous catheters further aggravate cancer-associated thrombosis (CAT) [2]. However, the treatment of CAT is challenging due to its high risk of recurrence and bleeding caused by anticoagulant therapy [3]. Although long-term parenteral therapy with low-molecular-weight heparin (LMWH) has been the standard management strategy for CAT for many years, and direct oral anticoagulants (DOACs) are receiving increasing attention, there is a need for more effective, safe, and convenient options for the treatment of CAT [4,5].

The pathophysiology of thrombosis in cancer is complex, and its regulatory mechanism remains to be elucidated. Tissue factor (TF) is a 47-kDa transmembrane glycoprotein that activates factor VII, and the TF-FVIIa complex serves as the initiator of coagulation reactions [6]. TF is extensively overexpressed in cancer and plays an important role in cancer-associated thrombosis, and has received immense attention recently [7]. The TF-positive microvesicles released by tumors are responsible for cancer-associated thrombosis in certain cancer types, including pancreatic and ovarian cancers [8,9,10]. However, little is known about the role of TF in breast cancer-associated thrombosis. The mechanisms by which tumors mediate the tissue factor expression in different cell types are largely unknown.

Neutrophils are the most abundant immune cells that act as the first line of defense against infection and injury in mammals. Neutrophil counts in peripheral blood are frequently increased in patients with cancer, especially in those at advanced stages, which are generally associated with poor clinical outcome [11]. Demers et al. showed an increase in peripheral neutrophils in a mammary carcinoma model, in which the von Willebrand Factor (VWF)- and fibrin-rich thrombi were found in the lungs of all 28-d tumor-bearing mice [12], suggesting a key role for neutrophils in facilitating cancer-associated thrombosis. Recent studies have reported that cancer cells predispose neutrophils to generate neutrophil extracellular traps (NETs), which are web-like structures that help to capture platelets and increase TF activity by binding elastase and cathepsin G, thus contributing to cancer-associated thrombosis [13,14,15,16]. However, the underlying mechanisms by which cancer stimulates neutrophils to generate NETs are unclear.

Both intracellular NAD^+^ and NADH levels are the fundamental metabolic regulators of cellular homeostasis and energy metabolism. Recent findings suggest that a disturbance of intracellular NAD^+^ levels is clinically related to the progression of many diseases, and maintenance of optimal intracellular NAD^+^ levels may be a critical factor for the prevention and treatment of these diseases. Approaches aimed at increasing the NAD^+^ levels by supplementing NAD^+^ precursors through the activation of de novo and salvage pathways for NAD^+^ biosynthesis have demonstrated cytoprotective effects against cellular damages. In addition to de novo and salvage NAD^+^ biosynthesis pathways as sources of cellular NAD^+^, the NAD(P)H quinone oxidoreductase 1 (NQO1) pathway also plays an important role in regulating cellular NAD^+^ levels [17]. NQO1 is an antioxidant flavoprotein enzyme that catalyzes the reduction and detoxification of quinones to hydroquinones by utilizing both NADH and NADPH as electron donors, which thus increases cellular NAD^+^ levels [18]. Dunnione, an orally administered substrate of NAD(P)H quinone oxidoreductase 1 (NQO1), is reported to increase cellular NAD^+^ levels in multiple tissues by accelerating the oxidation of NADH to NAD^+^, and has beneficial effects on various diseases, such as acute pancreatitis, cisplatin-induced tissue injury, and adriamycin-induced cardiac dysfunction [19,20,21]. NAD^+^ acts as a cofactor for NAD^+^-dependent enzymes, including sirtuins (SIRTs) and poly (ADP-ribose) polymerases (PARPs). SIRT1 plays crucial roles in a variety of biological processes through the deacetylation of histones and other important transcriptional factors, such as the nuclear factor-kappa B (NF-κB), p53, and peroxisome proliferator-activated receptor-gamma coactivator 1-alpha (PGC1-α), etc [18,19]. Therefore, we hypothesized that the modulation of cellular NAD^+^ levels by dunnione could suppress the tissue factor expression by regulating the NF-κB activity and inhibiting NET generation by deacetylating and stabilizing the histones of neutrophils, thus inhibiting the coagulation cascade and attenuating thrombus formation in cancer. In the present study, we used a murine orthotopic 4T1 breast cancer model with multiple thrombi in the lungs at an advanced stage to identify the efficacy of dunnione in cancer-associated thrombosis.

## 2. Results

### 2.1. Dunnione Suppresses Pulmonary Thrombosis in Advanced 4T1 Tumor Bearing Mice

**Figure 1:**
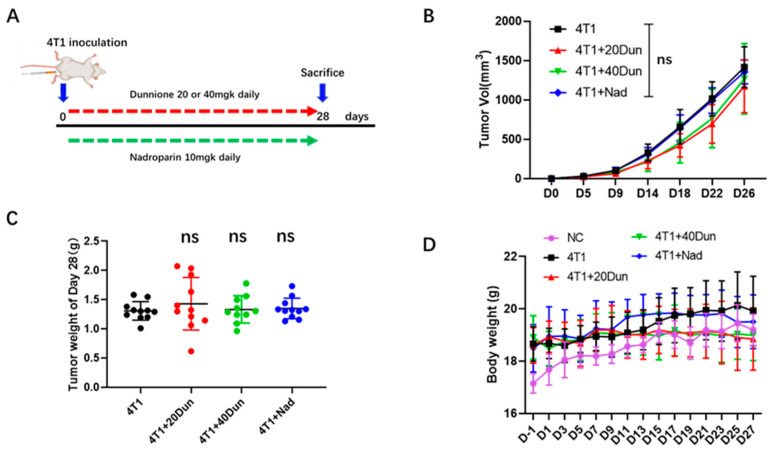

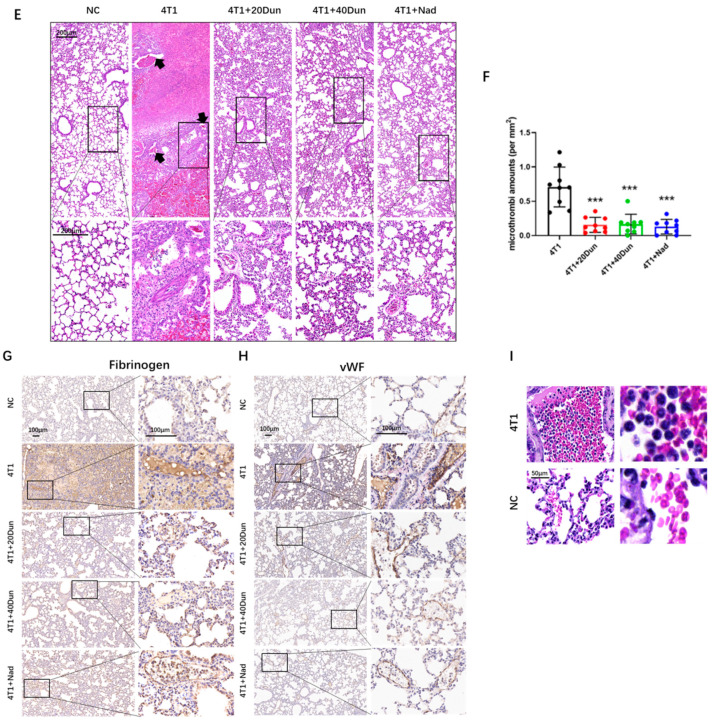
Dunnione inhibits 4T1 breast cancer-associated pulmonary thrombosis. (**A**) Diagram depicting the treatment schedule for the 4T1 breast cancer-associated thrombosis mouse model. Dunnione (dissolved in corn oil) and nadroparin were administrated daily, orally and subcutaneously. (**B**) Volume changes of orthotopically inoculated tumors in each group on the day of tumor injection (*n* = 8–11 per group). (**C**) Comparison of tumor weight of mice 28 d after tumor inoculation (*n* = 10–11 per group). (**D**) Body weight changes in the tumor-bearing mice and normal control mice in each group. Weight was measured every other day. (**E**) Representative hematoxylin and eosin (**E**,**H**) staining images of lung sections in each group. Black arrows indicate the thrombi (*n* = 8–11 per group). Scale bar, 200 μm. (**F**) Microthrombi counts per mm^2^ of lung section of each group at 28 d after tumor inoculation (*n* = 9 per group). (**G**,**H**) Representative immunohistochemistry (IHC) images of fibrinogen (**G**) and von Willebrand Factor (Vwf) (**H**) in the lung sections of each group (*n* = 4). Scale bar, 100 μm. (**I**) Comparison of pulmonary vessels by H&E staining in 4T1 tumor-bearing mice and normal mice. Scale bar, 50 μm. NC (normal control): tumor-free mice. *p*-values were obtained by one-way analysis of variance (ANOVA). Data represent the mean ± standard deviation (SD). Significance as compared to the samples of the 4T1 group. ns, not significant; *** *p* < 0.001.

First, a cancer-associated thrombosis mouse model was established according to a previous study in which female BALB/c mice were orthotopically injected with 4T1 breast cancer cells, and pulmonary thrombosis developed in mice at 28-d post implantation in the advanced stages of the disease [12]. Mice were then treated with dunnione (20 mg/kg), dunnione (40 mg/kg), or nadroparin (a type of LMWH, 10 mg/kg) starting on the day of cancer cell inoculation to investigate the effect of dunnione on breast cancer-associated pulmonary thrombosis (Figure 1A). Histologically, compared with tumor-free mice (NC, normal control group), the lungs of tumor-bearing mice exhibited thickened alveolar septum, obvious interstitial fibrosis, intra-alveolar hemorrhage with a large number of red blood cells and inflammatory cells in the bronchial and alveolar lumens, and multiple thromboses were observed in both venous and arterial lumens (Figure 1E). Remarkably, oral dunnione or subcutaneous nadroparin had no effect on primary tumor growth (Figure 1B,C), but obviously inhibited pulmonary thrombosis in tumor-bearing mice (Figure 1E). The overall changes in body weight were not significantly different among the groups, indicating no overt toxicity of treatment (Figure 1D). Hematoxylin and eosin staining revealed reduced counts of pulmonary microthrombi (arrows in Figure 1E) in the lungs of dunnione- or nadroparin-treated 4T1 tumor-bearing mice (Figure 1F). In addition, immunohistochemical staining showed significantly elevated expression of fibrinogen and vWF in the vessels and interstitium of lungs in 4T1 tumor-bearing mice compared with normal control mice, but were significantly attenuated by dunnione and nadroparin (Figure 1G,H). Furthermore, a marked increase in neutrophils was observed in the pulmonary vessels of 4T1 tumor-bearing mice (Figure 1I), suggesting that neutrophils may play a role in 4T1 breast cancer-associated pulmonary thrombosis formation. Together, these results demonstrate that dunnione inhibits pulmonary thrombosis in advanced 4T1 breast cancer and suggests a pro-thrombotic role of neutrophils in 4T1 breast cancer mice.

### 2.2. 4T1 Tumor-Bearing Mice Exhibit Neutrophilia and Increasing NETs, Which Could Be Attenuated by Dunnione

**Figure 2:**
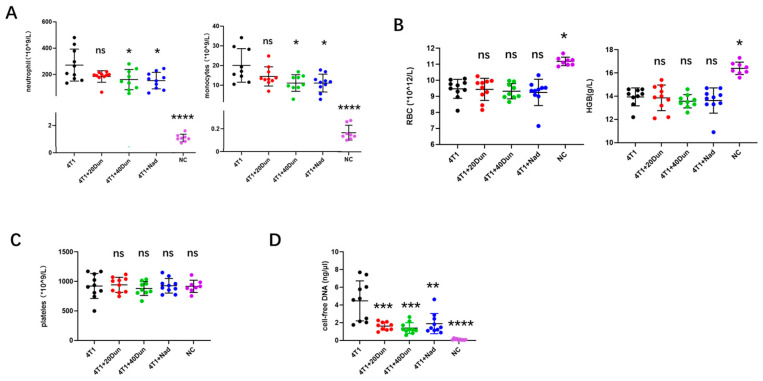

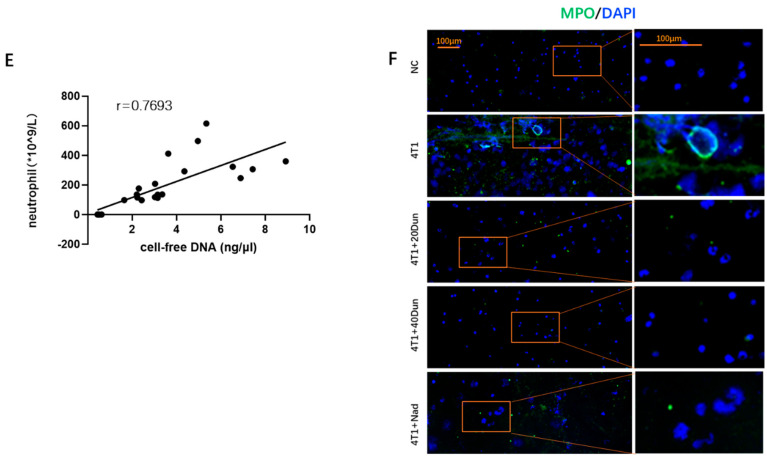
Dunnione alleviates neutrophilia and increases neutrophil extracellular traps (NETs) in 4T1 tumor−bearing mice. (**A**) Peripheral neutrophil and monocyte counts were measured by an automated hematology analyzer in each group (*n* = 8−11 per group). (**B**) Comparison of red blood cell (RBC) and hemoglobin (HGB) levels in each group (*n* = 8−11 per group). (**C**) Comparison of platelet counts in each group (*n* = 8−11 per group). (**D**) Systemic levels of cell−free DNA were detected by Quant−iT PicoGreen dsDNA kit in the plasma of each group (*n* = 8−10 per group). (**E**) Correlation of peripheral neutrophil counts and plasma cell−free DNA in mice of all groups (*n* = 24). (**F**) Myeloperoxidase (MPO) (green) combined with 4′, 6−diamidino−2−phenylindole (DAPI) (blue) staining of neutrophils isolated from the mice in each group (*n* = 4). Neutrophils were simulated with 500 ng/mL lipopolysaccharide (LPS) of for 30 min after isolation, and then slides were prepared by cytospin. Scale bar, 100 μm. *p*-values were obtained by one−way ANOVA. Data represent the mean ± SD. Significance as compared to the samples of the 4T1 group. ns, not significant; * *p* < 0.05; ** *p* < 0.01; *** *p* < 0.001; **** *p* < 0.0001.

In a variety of human cancers and murine solid tumor models, an increase in peripheral leukocytes is frequently observed and associated with poor clinical outcomes and a high risk of venous thromboembolism [11,20]. Because all mice inoculated with 4T1 mammary carcinoma cells without any treatment developed lung thrombosis at an advanced stage in our murine model, we wondered whether neutrophils play a major role in this cancer-associated thrombosis model and whether dunnione alleviates pulmonary thrombosis by regulating neutrophils. As a result, a leukemoid reaction was observed in tumor-bearing mice, with a dramatic increase in peripheral blood neutrophils and monocytes compared with normal control mice, which was significantly reduced by 40 mg/kg dunnione and nadroparin (Figure 2A). 4T1 tumor-bearing mice exhibited mild to moderate anemia with decreased erythrocyte and hemoglobin levels in peripheral blood compared with tumor-free mice, but did not respond to dunnione or nadroparin (Figure 2B). No significant differences were found in other hematologic components, such as platelets, among the groups (Figure 2C). Plasma analysis revealed a significant increase in cell-free DNA in 4T1 tumor-bearing mice compared with the controls, which was also reduced by treatment with dunnione and nadroparin (Figure 2D). We performed regression analysis as well, where a positive correlation between the level of peripheral neutrophil count and cell-free DNA (correlation coefficient r = 0.7693) was observed (Figure 2E), indicating that NETs from increasing neutrophils might be the major source of cell-free DNA. To determine whether tumors predisposed neutrophils to generate NETosis (NET formation) and whether dunnione could reduce NETosis, we treated the isolated neutrophils from mice of each group with low-dose lipopolysaccharide (LPS) (500 ng/mL) and observed the generation of NETs. Interestingly, low-dose LPS-treated neutrophils from 4T1-tumor-bearing mice presented a significant increase in NETs (Figure 2F) compared with the controls, which was remarkably reduced by dunnione and nadroparin, and even NETosis was observed without stimulation of LPS in tumor-bearing mice (Figure S1), suggesting that cancers predispose neutrophils to generate NETs, whereas dunnione could prevent NET formation in vivo. Taken together, our results suggest that tumors facilitate a prothrombotic state in the lungs of mice partially by stimulating neutrophilic leukocytosis and NET generation, whereas they were remarkably reduced by dunnione and nadroparin.

### 2.3. Dunnione Attenuates Neutrophilia in 4T1 Tumor-Bearing Mice by Regulating the Granulocyte Colony-Stimulating Factor (G-CSF) Rather Than the Granulocyte-Macrophage Colony-Stimulating Factor (GM-CSF)

**Figure 3:**
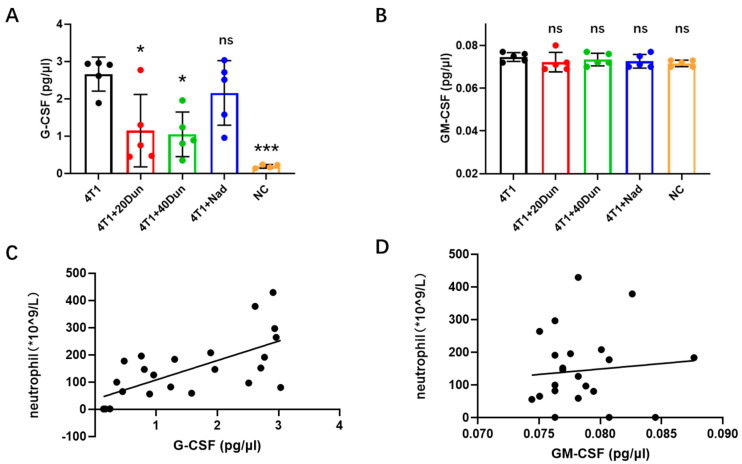
Dunnione reduces the peripheral neutrophil counts in tumor−bearing mice by modulating the granulocyte colony−stimulating factor (G−CSF) rather than the granulocyte−macrophage colony−stimulating factor (GM−CSF). (**A**,**B**) Systemic levels of G−CSF (**A**) and GM−CSF (**B**) were assessed by specific enzyme−linked immunosorbent assay (ELISA) kits following the manufacturer’s instructions (*n* = 5 per group). (**C**,**D**) Correlation of neutrophil counts to the plasma G−CSF levels (**C**) and GM−CSF levels (**D**) (*n* = 24). *p*−values were obtained by one-way ANOVA. Data represent mean the ± SD. Significances as compared to the samples of the 4T1 group. ns, not significant; * *p* < 0.05; *** *p* < 0.001.

Neutrophils are abundant leukocytes in both humans and mice, with a short half-life of 6–8 h in the circulation [21]. They require a constant replenishment and release from bone marrow to maintain homeostasis, which is tightly regulated by several mediators, particularly G-CSF and GM-CSF expressed by bone marrow stromal cells [22]. In 4T1 tumor-bearing mice, aberrant hematopoiesis was observed with extremely elevated peripheral neutrophils and monocytes, suggesting increased production of G-CSF and GM-CSF. Concordant with the results of neutrophil count in peripheral blood, plasma G-CSF levels in 28d- 4T1 tumor-bearing mice were significantly increased compared with tumor-free mice, which were significantly reduced by dunnione as well, and slightly reduced by nadroparin (Figure 3A). However, the expression of GM-CSF in the plasma was very low, and there were no significant differences among the groups (Figure 3B). Regression analysis was performed to determine the correlation between circulating neutrophils and the levels of G-CSF or GM-CSF. A strong correlation was observed between circulating neutrophils and G-CSF expression in the plasma (Figure 3C). In contrast, there was no correlation between circulating neutrophils and GM-CSF expression in plasma (Figure 3D). These data suggest that dunnione alleviated neutrophilia in 4T1 tumor-bearing mice by regulating G-CSF rather than GM-CSF.

### 2.4. Dunnione Ameliorates Cancer-Associated Thrombosis by Regulating the NAD^+^/SIRT1/acetyl-NF-kB/Tissue Factor Axis in the Lungs

**Figure 4:**
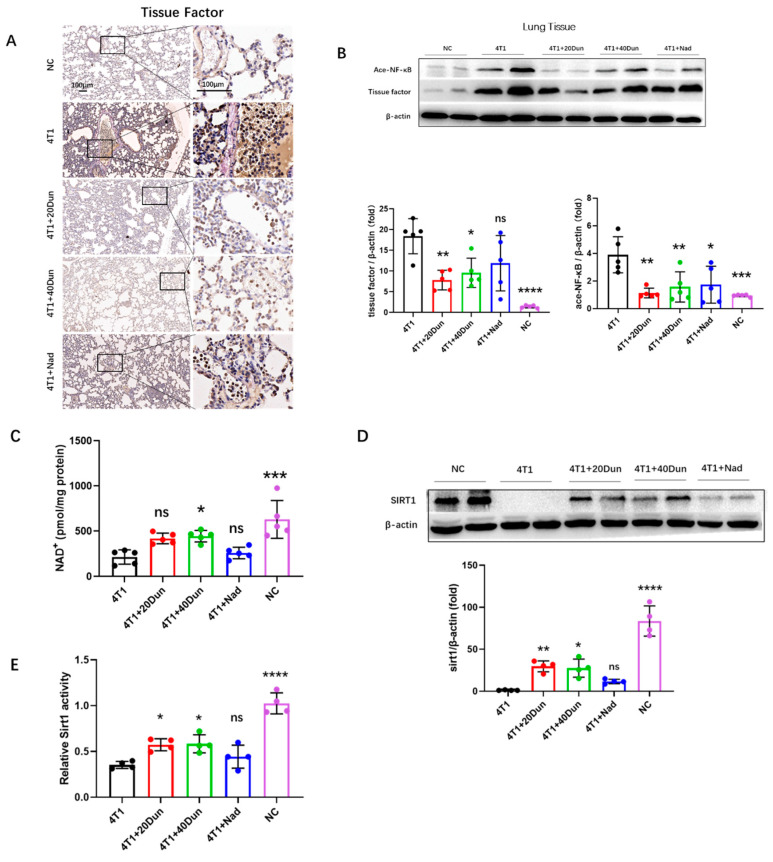
Dunnione attenuates breast cancer-associated lung thrombosis via the NAD(P)H quinone oxidoreductase 1 (NQO1) −nicotinamide adenine dinucleotide (NAD) −sirtuin 1 (SIRT1) −nuclear factor−kappa B (NF-kB) −tissue factor axis in vivo. (**A**) Representative IHC images for tissue factor staining of the lung sections in each group. Scale bar, 100 μm. (**B**,**D**) Western blotting analysis of acetyl-NF-κB, tissue factor (**B**), and SIRT1 (**D**) expression in lung tissues (*n* = 4 for SIRT1, others 5 per group). Densitometric analysis is presented as the fold induction of the target protein relative to β-actin. (**C**) NAD^+^ levels in lung tissues were assessed by using the NAD^+^/NADH assay kit in the mice of each group (*n* = 5 per group). (**E**) SIRT1 activity in lung tissues was measured using the SIRT1 activity assay kit (*n* = 4 per group). Data represent the mean ± SD. *p*-values were obtained by one-way ANOVA. Significance as compared to the samples of the 4T1 group. ns, not significant; * *p* < 0.05; ** *p* < 0.01; *** *p* < 0.001; **** *p* < 0.0001.

Next, we investigated the effect of dunnione on the formation of pulmonary thrombosis in 4T1 tumor-bearing mice. Because the transmembrane glycoprotein tissue factor (TF) is the prime initiator of the extrinsic coagulation cascade, and previous studies have reported that TF plays an essential role in cancer-associated thrombosis [8], we suggest that TF might promote pulmonary thromboembolism in 4T1 tumor-bearing mice. Concordantly, immunohistochemical staining showed obviously higher TF expression throughout the lung lobe of 28d 4T1 tumor-bearing mice compared with normal control mice (Figure 4A). Localization showed that TF was increased not only in pulmonary epithelial cells and intravascular thrombosis sites, but also in vascular endothelium, suggesting activation and injury of endothelial cells in tumor-bearing mice (Figure 4A). Remarkably, the overexpression of TF was significantly attenuated by dunnione (Figure 4A), and western blot analyses demonstrated similar results (Figure 4B). Furthermore, we investigated the molecular mechanism by which dunnione affects TF expression. Recent studies have reported that dunnione plays an important biological role by increasing cellular NAD^+^ levels and activities of NAD^+^-dependent enzymes, and has beneficial effects on multiple diseases, such as acute pancreatitis, adriamycin-induced cardiac dysfunction, and cisplatin-induced tissue injury [23,24,25,26]. Next, we examined NAD^+^ levels and SIRT1 expression in lung tissues. We found that NAD^+^ and SIRT1 expression was significantly reduced in the lung tissues of 4T1 tumor-bearing mice compared with that in normal mice, but was restored by dunnione (Figure 4C,D). Similarly, SIRT1 enzyme activity was significantly diminished in tumor-bearing mice compared to that in normal mice, which was also increased in the dunnione-treated groups (Figure 4F). Because tissue factor is a well-known downstream target of NF-κB [27], we asked whether dunnione decreased TF expression by regulating the SIRT1/acetyl-NF-κB/tissue factor axis. Consistently, ace-NF-κB expression in lung tissues was significantly higher in 4T1 tumor-bearing mice than in tumor-free mice, and was suppressed by dunnione (Figure 4B). In addition, we also found that dunnione reduced acetylated NF-κB and tissue factor expression in 4T1 tumor cells (Figure S2A–C). Collectively, these data suggest that dunnione alleviates breast cancer-associated lung thrombosis by inhibiting the expression of tissue factor through the NAD^+^/SIRT1/acetyl-NF-κB/tissue factor axis in vivo.

### 2.5. Dunnione Attenuates Tumor Cells-Induced NETs by Inhibiting Histone Acetylation and NOX Activity of Neutrophils

**Figure 5:**
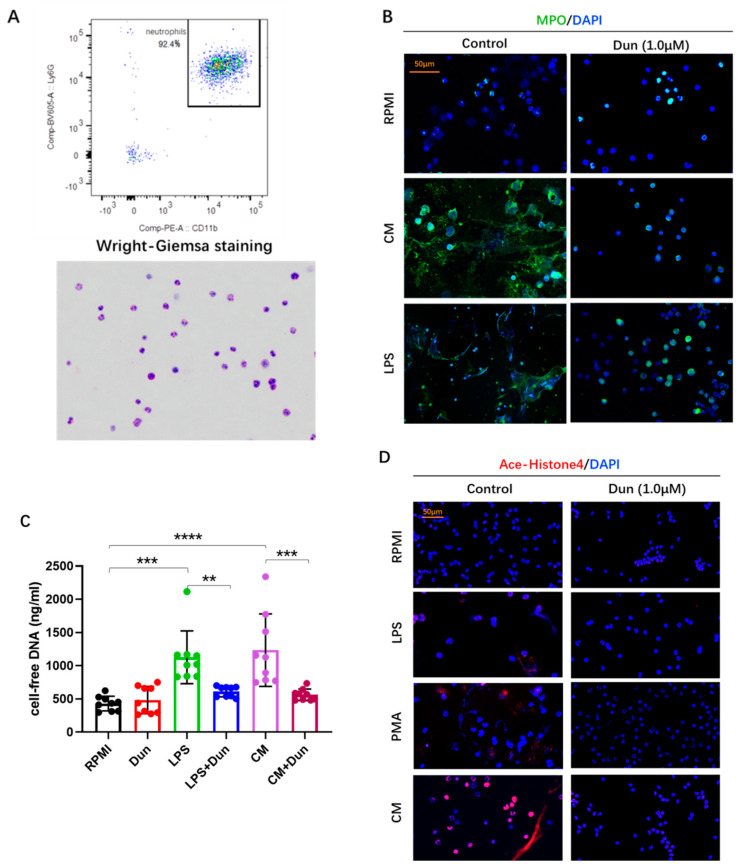

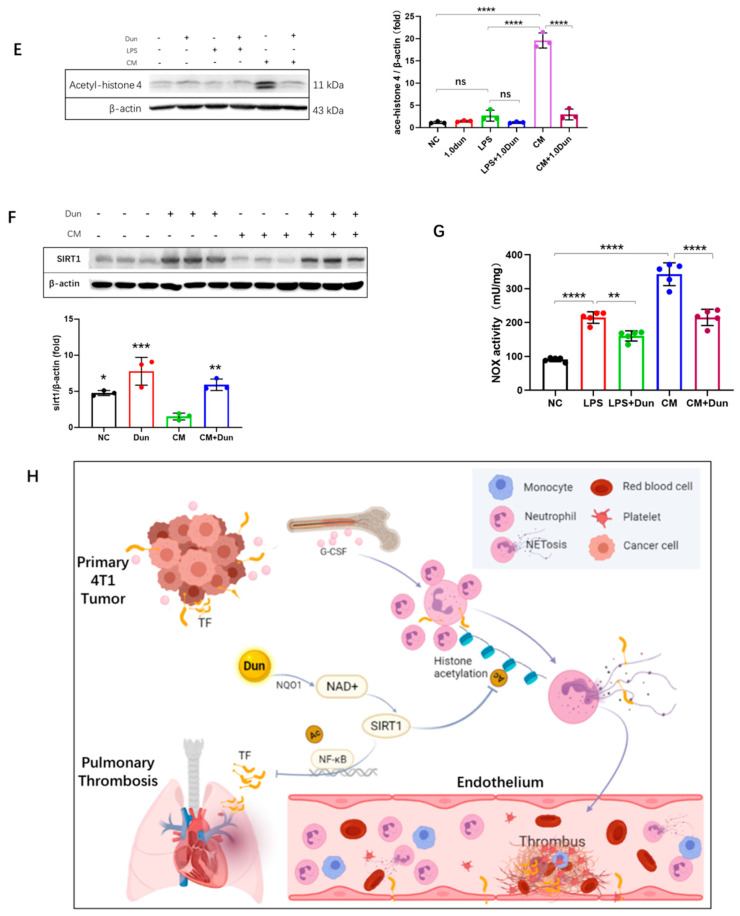
Tumor cells increase the formation of NETs by promoting histone acetylation and NOX activity of neutrophils, which could be attenuated by dunnione. Neutrophils were co−treated with the negative control (Roswell Park Memorial Institute (RPMI) medium), cancer cells conditioned medium, LPS (25 μg/mL) or phorbol myristate acetate (PMA) (5 μM) and dunnione (1.0 μM) for 3 h. (**A**) Flow cytometry and Wright−Giemsa staining to identify the purity of Percoll isolated neutrophils from the bone marrow of normal healthy mice. (**B**) MPO (green) immunostaining with DAPI (blue) counterstain in isolated neutrophils. Scale bar, 50 μm. (**C**) Quantification of cell−free DNA in supernatants of cultured neutrophils by Quant−iT PicoGreen dsDNA kit (*n* = 9 per group). (**D**) Acetyl-Histone 4 (red) combined with DAPI (blue) immunostaining of neutrophils from each treated group. Scale bar, 50 μm. (**E**,**F**) Western blotting analysis of acetyl−histone 4 (**E**) and SIRT1 (**F**) in neutrophils (*n* = 3 per group). Densitometric analysis is presented as the fold induction of acetyl−histone 4 or SIRT1 relative to β−actin. Significances of SIRT1 expression as compared to the negative control samples. (**G**) NOX activity in neutrophils was measured using an NADH oxidase activity assay kit according to the manufacturer’s instructions. (**H**) An overview schematic illustrating the regulatory effects of dunnione on cancer-associated lung thrombosis. Data represent the mean ± SD. *p*−values were obtained by one−way ANOVA. ns, not significant; * *p* < 0.05; ** *p* < 0.01; *** *p* < 0.001; **** *p* < 0.0001.

To investigate how tumors promote NET generation and the effect of dunnione on NETosis, isolated bone marrow neutrophils with a viability > 98% and purity > 90% (Figure 5A) were treated with 4T1 conditioned medium (CM) or LPS with or without dunnione. Consequently, we found that neutrophils formed abundant NETs when cultured in CM or stimulated with LPS, manifested as co-localization of extracellular DNA (blue) with MPO (green) by IF staining, which is considered to be a marker of NETosis (Figure 5B). However, NETosis induced by CM or LPS was obviously inhibited by dunnione, characterized by normal neutrophil morphology with multi-lobulated nuclei, similar to neutrophils cultured in the Roswell Park Memorial Institute (RPMI) (control) medium (Figure 5B). Measurement of extracellular DNA (pico green-stainable DNA) in the supernatant of cell culture also confirmed the effect of dunnione on the inhibition of NETosis. Incubating neutrophils with LPS or CM for 3 h demonstrated a marked increase in Pico Green stained DNA, but was significantly decreased when co-incubated with dunnione (Figure 5C).

Histone citrullination, which facilitates chromatin decondensation, is considered a hallmark of NETosis processing [28]. However, another post-translational modification, acetylation, could also decondense histones, resulting in chromatin relaxation [29]. Hussein et al. demonstrated that histone acetylation promotes NET formation in vitro [30]. Thus, we investigated whether histone acetylation plays a role in facilitating NETosis. Interestingly, we observed that acetylated histone 4 (AcH4) levels were significantly higher in neutrophils cultured in CM, whereas they were barely noticed in neutrophils that were untreated, treated with LPS and phorbol myristate acetate (PMA), or co-treated with dunnione (Figure 5D). Western blots showed similar results: neutrophils incubated with CM evoked increased AcH4 expression compared with other groups, which was also reduced by dunnione (Figure 5E). SIRT1 is a well-known NAD^+^-dependent histone deacetylase that plays essential roles in the deacetylation of histones [31]. In our experiments, dunnione significantly increased SIRT1 levels, which were decreased in neutrophils cultured with cancer CM (Figure 5F), suggesting that dunnione alleviates histone acetylation by increasing SIRT1 expression, which as a result reduces cancer-induced NETosis.

NOX-dependent and NOX-independent are two well-known major pathways during NET formation, which require NOX-derived reactive oxygen species (ROS) and mitochondrial ROS, respectively [32]. A previous study reported that dunnione ameliorates acute pancreatitis by inhibiting NOX activity and NOX-induced ROS production [26]. Therefore, we hypothesized that dunnione decreases CM- or LPS-stimulated NETosis by regulating the NOX activity of neutrophils. As shown in Figure 5G, neutrophils stimulated with CM and LPS exhibited increased NOX activity compared with the untreated controls, which was significantly reduced by dunnione treatment. These results suggest that tumor cells increase NET formation by promoting histone acetylation and NOX activity of neutrophils, which was attenuated by dunnione.

## 3. Discussion

Emerging evidence indicates that neutrophils and neutrophil extracellular traps play essential roles in thrombogenesis, especially in cancer-associated thrombosis, because neutrophils are frequently remodeled with an increasing amount and are more prone to generate NETs in cancers [12,13,14,15,33]. 4T1, a breast cancer cell line that is usually used in animal models to mimic stage IV human breast cancer, has recently been reported to trigger a dramatic increase in circulating neutrophils and extracellular DNA traps in 4T1 tumor-bearing mice [12,34,35]. In this study, we successfully established a murine breast cancer-associated thrombosis model through orthotopic injection of 4T1 cells, in which multiple lung thromboses were observed in advanced stages of cancer. In addition, we demonstrated that the NQO1 substrate dunnione attenuated pulmonary thrombosis in 4T1 tumor-bearing mice through the regulation of intracellular NAD^+^ levels. We explored the inhibition of NET formation and tissue factor as two mechanisms through which dunnione alleviated hypercoagulability and pulmonary thromboembolism in 4T1 tumor-bearing mice.

NETosis, by which neutrophils release web-like structures consisting of granular proteins and decondensed chromatin to trap and kill invading pathogens, was originally reported as a novel antimicrobial strategy of our host defense [36]. However, excessive NET formation at the wrong time or in the wrong place may contribute to a variety of diseases, such as sepsis, cancer metastasis, autoimmune disease, and thrombosis [15,35,37,38,39]. Recently, NETs have been identified as potential culprits of COVID-19 related pulmonary dysfunction and thromboembolism [40,41,42]. Our results demonstrated that cancers predisposed neutrophils to generate NETs related to cancer-associated thrombosis both in vivo and in vitro, whereas they were markedly inhibited by dunnione. In accordance with LPS and PMA, cancer cell-conditioned medium can also lead to NETosis, which is caused by tumor-derived chemokines. However, recent studies have shown that other mechanisms are involved in tumor-induced NETosis [43]. Interestingly, we found that tumors induced histone hyper-acetylation in neutrophils, which may contribute to chromatin relaxation and promote NET formation, suggesting that histone acetylation is a potential therapeutic target for tumor-NET-related diseases, such as cancer metastasis and cancer-associated thrombosis. Consistently, dunnione significantly suppressed NETosis by increasing SIRT1 activity and deacetylating neutrophil histones. Furthermore, because NOX activation is critical for NOX-dependent NETosis [32], we investigated the effect of dunnione on the NOX activity of neutrophils during NETosis. Previously, our laboratory showed that dunnione ameliorated acute pancreatitis by regulating NOX activity and ROS production [26], and here we also found that dunnione significantly reduced NOX activity in isolated neutrophils, which was increased during NETosis.

Tissue factor (TF), a transmembrane glycoprotein that acts as the primary initiator of the coagulation cascade, which is universally overexpressed in cancer, has been intensively investigated in cancer-associated thrombosis [8]. In this study, we found that dunnione effectively reduced the expression of tissue factor in lung tissues through the NAD^+^/SIRT1/acetyl-NF-kB/tissue factor axis. NAD^+^ is a coenzyme in multiple redox reactions and plays an essential role in energy metabolism, such as glycolysis, oxidative phosphorylation, and the tricarboxylic acid cycle [44]. In addition, NAD^+^ also acts as a substrate for a variety of enzymes, including SIRTs and poly ADPribose polymerases (PARPs), which are involved in many biological processes, such as protein deacetylation and DNA damage repair [44]. We found that NAD^+^ level and SIRT1 were significantly decreased in the lungs of tumor-bearing mice compared with normal mice. Dunnione restored reduced SIRT1 activity in lung tissues of tumor-bearing mice by augmentation of NAD^+^, thus deacetylating NF-κB and reducing NF-κB activity, resulting in decreased TF expression and activity. Immunohistochemical localization of TF in lung tissues revealed that elevated TF was not only observed in epithelial cells, which are the primary site, but also in circulating cells, including neutrophils and monocytes. Recent studies reported that TF derived from neutrophils could be specifically exposed by NETs, and NET-bound TF showed strong thrombogenic activity in atherothrombosis and acute myocardial infarction [45,46]. However, the interaction between NETs and TF in cancer-associated thrombosis has not been fully elucidated, suggesting that severing the link between NETs and TF may be a potential novel strategy for the treatment of cancer-associated thrombosis.

However, dunnione had no effect on primary tumor growth in our orthotopic 4T1 breast cancer mouse model. We suppose the possible reason is that drug distribution within the breast cancer is affected by both drug (physicochemical characteristics) and tumor microenvironment including blood vessels, lymphatic vasculature, extracellular matrix composition and interstitium [47]. Within the solid tumor, blood vessels are highly heterogeneous and usually not normal-organized, resulting in an obviously increased interstitial fluid pressure that could hamper the diffusion of drugs [47]. In these critical conditions, we assume that, unlike the ease with which it diffuses to lung, it is difficult for dunnione, a lipophilic drug that is administrated orally and absorbed through intestinal blood vessels, to diffuse into the tumor and form a sufficient concentration, thus resulting in a reduced anti-tumor activity.

Overall, our results demonstrated that dunnione effectively prevented cancer-associated hypercoagulability and attenuated thrombosis by inhibiting tumor-derived tissue factor and neutrophil extracellular trap formation via the regulation of cellular NAD^+^ levels. In addition, we found that the histone acetylation of neutrophils may be a novel mechanism by which cancer cells trigger NET formation to promote cancer-associated thrombosis.

## 4. Materials and Methods

### 4.1. Animals

All animal procedures were performed using 8–9 weeks old BALB/c female mice, weighing 19 g, purchased from the Central Laboratory Animal Inc. (Seoul, Korea). Mice were housed at an ambient temperature of 20–22 °C under a 12/12 h light/dark cycle in a specific pathogen-free facility. All experimental procedures were conducted in accordance with ethical standards and were approved by the Animal Care and Use Committee of Wonkwang University, Republic of Korea.

### 4.2. Cell Lines and Reagents

The 4T1 cell lines were cultured in the RPMI 1640 medium (11875-093; Gibco, UK) supplemented with 10% *v*/*v* fetal bovine serum (S001-07; Welgene) and 1× antibiotic antimycotic containing streptomycin and penicillin (15240-062; Gibco, USA) at 37 °C in a humidified atmosphere of 5% carbon dioxide (CO_2_). Cells were inoculated into mice after fewer than five serial passages in culture. Dunnione was chemically synthesized by Erum Biotechnologies (Suwon, Korea) and sonicated as particles using an ultrasonic cleaner (Power Sonic 510; Daejeon, Korea). Nadroparin calcium was purchased from Kingfriend (Nanjing, China).

### 4.3. Induction of Solid Tumors

The 4T1 cells (in 30 μL of RPMI medium with 1:1 Matrigel) were orthotopically inoculated into the mammary fat pad of the eight to nine weeks old BALB/c mice. Mice were weighed every other day, and the tumor volume was carefully measured twice a week using calipers. Tumor volume was calculated according to the formula V (volume, mm^3^) = lw^2^/2, where l is the tumor length (mm), and w is the width (mm). Mice were sacrificed at the indicated time (D28), and the resected tumors were weighed.

### 4.4. Blood Cell Analysis

At sacrifice, whole blood samples were collected in ethylenediaminetetraacetic acid (EDTA)-coated tubes. Blood cell counts were measured using an automated hematology analyzer (XN-1000; Sysmex; Kobe, Japan), where the qualitative flagging of immature white blood cells and fluorescent platelet technology were used to improve the accuracy and specificity of cell counts.

### 4.5. Isolation of Neutrophils

Six to eight week old healthy mice were euthanized following the animal care committee-approved protocol. Femurs and tibias were then removed and washed in ice-cold sterile RPMI 1640 medium three times. Both ends of the bone were cut off and the bone marrow cells were flushed with RPMI 1640 and filtered through a 70 μm filter. The cells were centrifuged for 5 min at 1500 rpm at 4 °C, resuspended in RPMI 1640 medium supplemented with 10 mM 4-(2-hydroxyethyl)-1-piperazineëthanesulfonic acid (HEPES) buffer (15630106; Gibco), laid on the top of 2-layer Percoll (P1644; Sigma-Aldrich; St Louis, USA) gradients of 78% and 65% in phosphate-buffered saline (PBS) (vol/vol), and then centrifuged at 800 g for 35 min at 25 °C, without any brake. Mature neutrophils were collected from 65–78% interface fractions, red blood cells were eliminated using ACK RBC lysis buffer (A10492-01; Gibco), and then washed two to three times with PBS to eliminate the RBC debris and soluble components. Cells were then stained with acridine orange/propidium iodide stain (F23001; Logos Biosystems) and measured using a fluorescence cell counter (LUNA-FL; Logos Biosystems), with a viability > 98%. The purity of the neutrophils used in the experiments was established to be >90% as assessed by Wright-Giemsa staining on cytospin slides (Cytospin 4; Thermo Scientific; Mass, USA) and flow cytometry (FACSymphony A3 flow cytometer; BD) with CD11b+Ly6G+ cells (anti-CD11b, 101208, BioLegend; anti-Ly6G, 563005, BD Biosciences, NJ, USA).

### 4.6. Quantification of the Plasma and Supernatant Cell Free-DNA

The quantification of cell-free DNA in plasma or supernatant of neutrophil culture was determined using the Quant-iT PicoGreen dsDNA kit (P11496; Invitrogen, USA) according to the manufacturer’s instructions. Briefly, 5–10 μL of the mouse plasma or supernatant of neutrophil cultures of different groups, as designed, was diluted in 90–95 μL Tris-EDTA (TE) buffer, and 100 μL Picogreen was added into each well in a Luminescence test 96-well plate. All samples were excited at 485 nm, and the fluorescence intensity was measured at 535 nm for emission using a multi-detection microplate reader (SpectraMax M3; Molecular Devices, USA). The experiments were repeated twice using different dilutions of samples to confirm the quantification results.

### 4.7. Induction of NETs In Vitro

For cancer cell conditioned medium (CM) collection, 4T1 cells were grown to approximately 80% in 10 cm culture dishes. After three washes with PBS, the cells were incubated in serum-free RPMI 1640 medium at 37 °C and 5% CO_2_ for 24 h. The CM was collected, centrifuged at 4000 rpm for 5 min, filtered with 0.22 μm filters, and stored at –20 °C until use. To induce the formation of NETs in vitro, an appropriate number of neutrophils were seeded on coverslips in 12-well or 96-well plates, and then 50% CM, LPS (L2630; Sigma-Aldrich), and PMA (P8139; Sigma-Aldrich) were added. After culturing for 3 h at 37 °C, the neutrophils were fixed for staining, and the supernatants were collected for cell-free DNA detection.

### 4.8. Immunostaining of NETs (Fluorescence Confocal Imaging)

After NET induction, neutrophils were fixed with 4% paraformaldehyde for 15 min at room temperature, washed three times with PBS, and then permeabilized in 0.1% Triton X-100 for 10 min. Next, the cells were blocked with PBS containing 5% bovine serum albumin (BSA) for 30 min, then incubated with anti-MPO (1:300, AF3667; R&D Systems, USA) and anti-acetylated histone 4 (1:300, ab51997; Abcam, UK) in a blocking buffer overnight at 4 °C. After washing three times with PBS, the cells were again incubated with fluorochrome-conjugated secondary antibodies (1:1000, green fluorescence Alexa Fluor 488 dye plus a red fluorescence Alexa Fluor 568 dye; Invitrogen) for 40 min at room temperature, and then counterstained with ProLong Gold antifade reagent with 4′, 6-diamidino-2-phenylindole (DAPI) (P36931; Invitrogen) before mounting. The cells were observed and photographed using a confocal microscope observer (ZEISS Axio Scan, Z1), and image processing and analysis were performed using the Zen blue edition software.

### 4.9. NADH Oxidase Activity

NADH oxidase activity in isolated neutrophils was measured using an NADH oxidase activity assay kit (K2028, Biovision, USA) according to the manufacturer’s instructions. The NOX assay kit couples the oxidation of NADH by NOX and the reduction of a blue substrate to a colorless product, which results in a decrease in absorbance measured at 600 nm. NOX activity in neutrophils was quantified by reading the absorbance changes at 600 nm for 30 min. The results are expressed as fold-change according to the protein amount.

### 4.10. Western Blotting Analysis

Lung tissues were homogenized with cold RIPA lysis buffer (LPS solution, Daejeon, Korea) using a tissue homogenizer (IKA, Homogenizer workcenter), and cultured neutrophils were collected using a scraper and then lysed with a cold RIPA lysis buffer on ice for 20 min, followed by centrifugation at 13,200 rpm for 20 min. Protein concentration was measured using a BCA Protein Assay Kit or Bio-Rad protein assay kit. Protein samples (10 μg for acetylated-histone 4 and 20 μg for the other assays, respectively) were separated on a 17 or 10% sodium dodecyl sulfate (SDS)-polyacrylamide gel for 90 min at 100v, and electrophoretically transferred to nitrocellulose membranes. The membranes were blocked with 5% BSA or dried milk protein in Tris-buffered saline (PBS) containing 0.05% Tween-20 (TBST) for 1 h, washed three times with TBST, and subsequently incubated with primary antibodies (anti-acetyl-NF-κB, PA5-17264, Invitrogen; anti-tissue factor, NBP2-67731, Novus Biologicals; anti-SIRT1, 07-131, Millipore; anti-acetyl-histone 4, ab51997, Abcam; 1:1000 dilution) at 4 °C overnight. The membranes were then incubated with a horseradish peroxidase (HRP)-conjugated secondary antibody (1:5000, A120-101P; Bethyl) for 1 h at room temperature. Protein intensity was determined using a western blot detection kit (LF-QC0103; YOUNG IN FRONTIER), and the bolts were imaged using a chemiluminescence imaging system (LuminoGraphII, ATTD), followed by densitometry analysis using a software of CSAnalyzer4. For β-actin detection, after the primary antibodies were washed out with stripping buffer, the membranes were incubated with HRP-conjugated β-actin antibody (1:1000, sc-4778; Santa Cruz Biotechnology) for 2 h at room temperature. The primary protein bands were normalized to the β-actin bands.

### 4.11. Quantification of G-CSF and GM-CSF in the Plasma

Specific enzyme-linked immunosorbent assay (ELISA) kits were used to assay the levels of mouse G-CSF (EMCSF3X5; Invitrogen) and GM-CSF (BMS612; Invitrogen) in the plasma according to the manufacturer’s instructions. Briefly, 96 well polystyrene microplates were coated with a polyclonal antibody specific for mouse G-CSF or GM-CSF. Then, 50 μL of Mouse plasma and standard samples were added to the wells and incubated for 2 h at room temperature on a shaker, and the plates were washed five times with wash buffer using an automated microplate washer (AquaMax 2000; Molecular Devices, Silicon Valley, USA). Next, 100 μL of mouse G-CSF or GM-CSF conjugate was added to the wells and incubated for 2 h at room temperature. After five washes with wash buffer, the substrate solution was added and incubated for 30 min at room temperature. Then, following the stop solution, the optical density of each well was determined using a microplate reader (SpectraMax M3; Molecular Devices) set to 450 nm within 30 min.

### 4.12. Lung Histology and Immunohistochemistry (IHC) Staining

One lobe of the right lung was harvested after the mice were sacrificed and fixed with 10% formaldehyde solution. Then, the paraffin-embedded sections were deparaffinized in xylene and rehydrated using a series of graded alcohols. The sections were then stained with hematoxylin and eosin (H&E), mounted with the HistoChoice mounting medium, and observed under a light microscope. An HRP-diaminobenzidine (HRP/DAB) detection IHC kit (ab64264; Abcam) was used to detect fibrinogen and vWF, according to the manufacturer’s instructions. Briefly, the sections were treated with the hydrogen peroxide block for 10 min, followed by application of protein block and incubation for 10 min at room temperature to block the non-specific background staining. Next, the sections were stained with a rabbit anti-fibrinogen antibody (1:500, ab34269; Abcam) and rabbit anti-vWF antibody (1:300, PA5-16634; Invitrogen), and incubated overnight at 4 °C. After being adequately washed in the PBS buffer four times, the sections were treated with biotinylated goat anti-polyvalent antibody and incubated for 10 min, followed by application of streptavidin peroxidase for 10 min at room temperature. Tissues were then applied with sufficient DAB mix of chromogen and substrate, incubated for 2 min, and washed in PBS. Nuclei were counterstained with haematoxylin. Finally, the sections were dehydrated, placed on coverslips, and observed under a light microscope (ZEISS Axio Scan, Oberkochen, Germany).

### 4.13. Measurement of NAD^+^ Level

NAD^+^ and NADH levels and their ratio were determined using an EnzyChrom NAD^+^/NADH assay kit (E2ND-100; BioAssay Systems, USA). Briefly, the tissues or cells were washed with cold PBS and homogenized in the NAD extraction buffer for NAD determination. Extracts were heated at 60 °C for 5 min and then neutralized by adding the NADH extraction buffer. After centrifugation at 14,000 rpm for 5 min, the supernatants were extracted for NAD/NADH assays. The optical density for time “zero” (OD_0_) reading at 565 nm of a working reagent mix containing 40 μL samples, 60 μL assay buffer, 1 μL enzyme A, 1 μL enzyme B, 14 μL lactate, and 14 μL 3-(4, 5-dimethylthiazol-2-yl)-2, 5-diphenyltetrazolium bromide (MTT) was taken immediately. OD_15_ was read after 15-min incubation at room temperature. Calculations were performed using an NAD(H) standard curve.

### 4.14. Determination of SIRT1 Activity

SIRT1 enzymatic activity was measured using the SIRT1 activity assay kit (ab156065; Abcam) according to the manufacturer’s instructions. Briefly, lung tissues were homogenized in a lysis buffer, and the whole protein was normalized according to the protein concentration. Then, the lysate, fluoro-substrate peptide, developer, and NAD were incubated at room temperature for 30 min. After stopping the reaction with the stop solution, the fluorescence intensity was measured using a microplate fluorescence reader (SpectraMax M3; Molecular Devices) at an excitation wavelength of 350 nm and an emission wavelength of 450 nm.

### 4.15. Statistical Analysis

Data analyses were performed using the GraphPad Prism software v.8.0, and all data are presented as the mean ± standard deviation (SD) of triplicate analyses. One-way analysis of variance (ANOVA) was used to analyze the statistical significance of the results, and *p* values < 0.05, were considered statistically significant. The animal experiments were performed at least three times, and the in vitro experiments were repeated independently multiple times with similar results.

## Data Availability

Not applicable.

## Supplementary Materials

The following are available online at https://www.mdpi.com/article/10.3390/ijms222112085/s1.
