# Supplementary material for: Modulation of Cellular NAD+ Attenuates Cancer-Associated Hypercoagulability and Thrombosis via the Inhibition of Tissue Factor and Formation of Neutrophil Extracellular Traps
Source: Int J Mol Sci. 2021 Nov 8;22(21):12085. doi: 10.3390/ijms222112085 (PMC8584923; doi:10.3390/ijms222112085)
Supplement: Supplementary file 1 [file ijms-22-12085-s001.zip › ijms-1412540-supplementary.pdf]

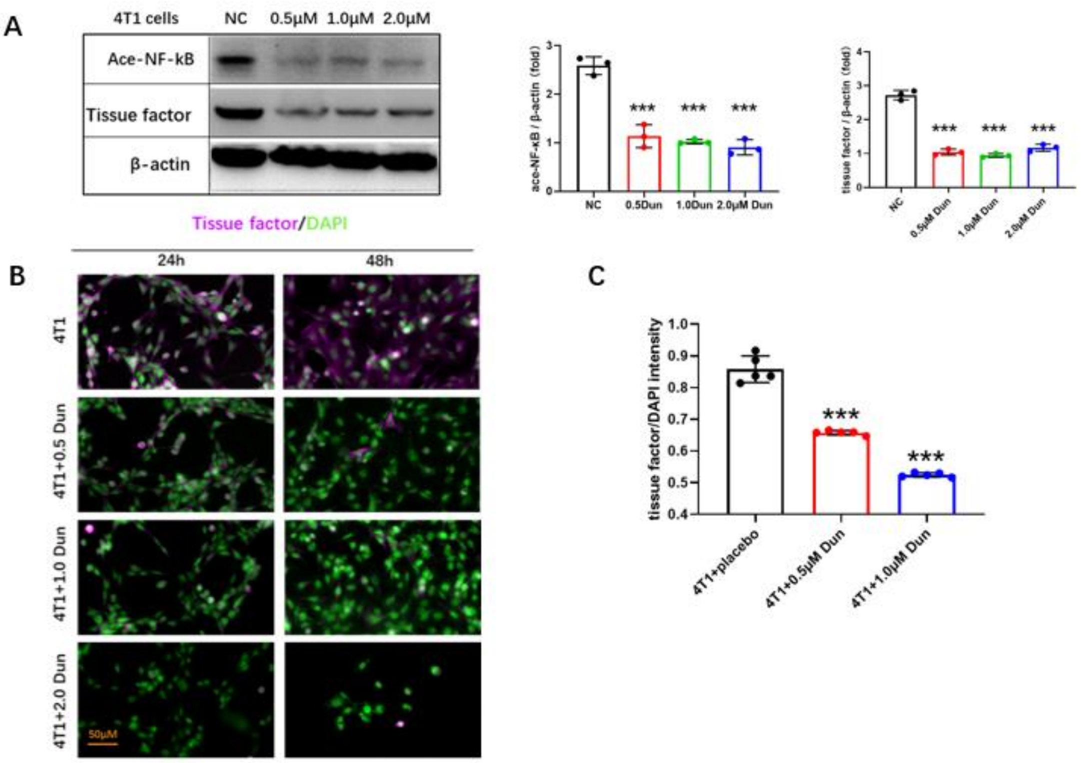

Figure S1. Dunnione decreases the tissue factor generated by 4T1 cells. (A) Western blotting analysis of the expression levels acetyl-NF-Kb and the tissue factor in 4T1 cells pretreated with 0.5 or 1.0 μM dunnione or placebo for 48 h (n = 3). Densitometric analysis is presented as the fold induction of the target protein relative to β-actin. (B) Tissue factor (purple) combined with DAPI (green) staining of 4T1 cells pre-treated with gradient (0.5, 1.0, and 2.0 μM) dunnione or placebo for 48 h. Scale bar, 50 μM. (C) The tissue factor/DAPI fluorescence intensity ratio was performed to assess the expression of tissue factor. n = 5 random microscopic fields from each group. Data represent the mean ± SD. P-values were obtained by one-way ANOVA. Significance as compared to the samples of the 4T1 + placebo group. ns, not significant; ***P < 0.001.
